# Ruminal pH pattern, fermentation characteristics and related bacteria in response to dietary live yeast (*Saccharomyces cerevisiae*) supplementation in beef cattle

**DOI:** 10.5713/ab.21.0200

**Published:** 2021-08-25

**Authors:** Xiangfei Zhang, Xianwen Dong, Metha Wanapat, Ali Mujtaba Shah, Xiaolin Luo, Quanhui Peng, Kun Kang, Rui Hu, Jiuqiang Guan, Zhisheng Wang

**Affiliations:** 1 Low Carbon Breeding Cattle and Safety Production-University Key Laboratory of Sichuan Province, Animal Nutrition Institute, Sichuan Agricultural University, Chengdu 611130, China; 2 Institute of Plateau Animals, Sichuan Academy of Grassland Science, Chengdu 611731, China; 3 Chongqing Academy of Animal Science, Chongqing 402460, China; 4 Tropical Feed Resources Research and Development Center, Department of Animal Science, Faculty of Agriculture, Khon Kaen University, Khon Kaen 40002, Thailand

**Keywords:** Beef Cattle, pH, Rumen Fermentation, Rumen Microorganism, *Saccharomyces Cerevisiae*, Serum

## Abstract

**Objective:**

In this study we aimed to evaluate the effect of dietary live yeast supplementation on ruminal pH pattern, fermentation characteristics and associated bacteria in beef cattle.

**Methods:**

This work comprised of *in vitro* and *in vivo* experiments. *In vitro* fermentation was conducted by incubating 0%, 0.05%, 0.075%, 0.1%, 0.125%, and 0.15% active dried yeast (*Saccharomyces cerevisiae*, ADY) with total mixed ration substrate to determine its dose effect. According to *in vitro* results, 0.1% ADY inclusion level was assigned in *in vivo* study for continuously monitoring ruminal fermentation characteristics and microbes. Six ruminally cannulated steers were randomly assigned to 2 treatments (Control and ADY supplementation) as two-period crossover design (30-day). Blood samples were harvested before-feeding and rumen fluid was sampled at 0, 3, 6, 9, and 12 h post-feeding on 30 d.

**Results:**

After 24 h *in vitro* fermentation, pH and gas production were increased at 0.1% ADY where ammonia nitrogen and microbial crude protein also displayed lowest and peak values, respectively. Acetate, butyrate and total volatile fatty acids concentrations heightened with increasing ADY doses and plateaued at high levels, while acetate to propionate ratio was decreased accordingly. In *in vivo* study, ruminal pH was increased with ADY supplementation that also elevated acetate and propionate. Conversely, ADY reduced lactate level by dampening *Streptococcus bovis* and inducing greater *Selenomonas ruminantium* and *Megasphaera elsdenii* populations involved in lactate utilization. The serum urea nitrogen decreased, whereas glucose, albumin and total protein concentrations were increased with ADY supplementation.

**Conclusion:**

The results demonstrated dietary ADY improved ruminal fermentation dose-dependently. The ruminal lactate reduction through modification of lactate metabolic bacteria could be an important reason for rumen pH stabilization induced by ADY. ADY supplementation offered a complementary probiotics strategy in improving gluconeogenesis and nitrogen metabolism of beef cattle, potentially resulted from optimized rumen pH and fermentation.

## INTRODUCTION

In recent decades, the rumen ecosystem of polygastric herbivores has been widely studied because of its critical role in feed efficiency, production and health. With the purpose of optimizing rumen environment, there were many attempts to develop new feeding strategies and the use of additives [1]. Probiotics, being defined as viable microorganisms, have a beneficial effect on animal health and have become one of the safe feeding additives following the restricted use of antibiotics. As a classified probiotic, yeast (*Saccharomyces cerevisiae* [*S. cerevisiae*]) can be formulated in ruminant rations on account of its advantage on improving productive performance [2]. Currently, commercial yeast products generally consist of yeast cultures, cellular extract or constituent and active dried yeast (ADY) which is otherwise known as live yeast cells [3]. Besides these products include cell components containing organics, minerals, amino acids, vitamins and yeast polysaccharides which are verified to be a superior nutritional source for autochthonous bacteria [4]. The live yeast cells can remain metabolically active in the ruminal ecosystem as well [5].

In the intensive beef industry, high readily fermentable carbohydrates with a low proportion of roughage are proposed to meet the energy requirements of production or fattening for high-producing ruminants and have obtained positive responses [6,7]. However, one of the negative consequences of a high concentration diet is the decrease of ruminal pH due to rapid short-chain fatty acids (SCFA) and lactate accumulations, which are fermented from non-structural carbohydrates by microbial community in the rumen [8,9]. Owing to the pH sensitivity of bacteria, failure of ruminal pH stabilization can cause activity suppression in microorganisms [10], and consequently lower feed utilization efficiency. Ruminal acidosis can occur with continuously low pH which worsens microflora disturbance and lesions in the gastrointestinal barrier [11]. Penner [12] summarized some evidence supporting the pH stabilizing effect of SCFA absorption. Oppositely, the decrease of ruminal pH was characterized by an accumulation of lactate concentration in the report of Luo et al [13]. Fortunately, in addition to benefits to production, *S. cerevisiae* was also proved to stabilize rumen pH and dry matter (DM) digestibility by Cagle et al [14], indicating a better microbial fermentation. But researches focusing on continuous monitoring and comparison between rumen volatile fatty acids (VFA) and lactic acid metabolism in the presence of live yeast, and alteration of lactate producing and consuming bacteria (*Streptococcus bovis*, *Lactobacillus* spp. and *Selenomonas ruminantium*, *Megasphaera elsdenii*) remain scarce. It rises the query of the pathway involved in stabilizing ruminal pH by live yeast supplementation. Whether it results from influencing metabolism of VFA or lactate through its interaction with related microbial community has also not been well established.

The studies on live *S. cerevisiae* supplementation in ruminant rations have shown increases in feed intake, feed efficiency, milk production enhancement and alleviating stress in lactating dairy cows [2,15]. But limited researches were conducted using beef cattle, particularly with respect to blood metabolic profile which can benefit from improved ruminal functions and indicates the process of metabolism and production. Beyond that, reports on impact of live yeast cells on rumen fermentation and microbes exhibited divergent results. No shift of rumen fermentation and bacterial population was illustrated by Magrin et al [16], on the contrary, Sousa et al [17] demonstrated different data implying ruminal fermentation improvement, stimulation of cellulytic microbes as well as pH stabilization. There is no consensus whether the discrepancy is derived from dose-dependent effect of yeast or not, and few researchers have focused on the question how active *S. cerevisiae* influence on ruminal VFA and lactic acid metabolism dynamically to maintain pH via its interaction with microbes. Therefore, our study aimed to explore the impact of ADY supplementation on ruminal pH pattern, fermentation characteristics, bacteria related to lactate metabolism and blood components in beef cattle.

## MATERIALS AND METHODS

### *In vitro* experiment

#### Experimental design and diets

This research was reviewed and approved by the Institutional Animal Care and Use Committee of Sichuan Agricultural University (YYS19021), and all procedures followed Animal Experimentation guidelines. *In vitro* fermentation study was carried out to evaluate the effect of gradient dietary ADY levels (*S. cerevisiae*, Angel Yeast Co., Ltd, Hubei, China, containing 2.0×10^10^ cells/g) on rumen microbial fermentation characteristics. Fermentation ration was dosed at six dietary ADY levels (0%, 0.05%, 0.075%, 0.1%, 0.125%, and 0.15% of substrate DM, respectively). The diet fed to ruminally fistulated beef cattle (461.32±18.94 kg) and *in vitro* fermentation substrates were consistent and formulated according to the Feeding Standard of Beef Cattle in China (2004), and concentration-to-roughage ratio was 63:37 (Table 1).

Three healthy ruminally cannulated Xuanhan Yellow cattle were used as donors of rumen fluid. After collection of rumen fluid, it was filtered through four layers of gauze and composited into flask which was placed in 39°C water-bath with sustained CO_2_ flushing. Artificial saliva was prepared referring to Menke et al [18]. The fermentation medium consisted of rumen fluid and artificial saliva in proportion of 1:2. Culture glass syringes containing 200 mg DM fermentation substrate with different ADY dosages were used for incubation. The mixed medium in volume of 30 mL was transferred into the glass syringes anaerobically with rubber hose at the top for seal. There were six units per treatment incubated at 39°C for 24 h with same shaking speed (n = 6).

#### Sampling and analysis

Cumulative gas production (GP) was recorded and corrected by blank control after *in vitro* incubation for 24 h. Aliquot *in vitro* incubation fluid of each unit was filtered and collected into 10 mL tubes at the end of fermentation, and pH value was immediately determined with pH meter (pHS-3D, Rex, Shanghai, China). All samples were then stored at −20°C for further analysis. NH_3_-N in the fermentation medium was detected according to the method from Searle [19]. Microbial crude protein (MCP) concentration was determined with a commercial reagent kit (BCA Protein Assay Kit (CAT#:80815-500), Tiandz Inc., Beijing, China) following manufacturer instruction. The VFA (including acetate, propionate, and butyrate) were measured via gas chromatograph (CP-3800GC, Varian, Walnut Creek, CA, USA). Fermentation fluid sample in volume of 1 mL was mixed with 0.2 mL metaphosphoric acid and centrifuged at 10,000 r/min for 15 min for VFA analysis. A flame ionization detector was used with an oven temperature of 200°C. The polyethylene glycol column was operated with highly purified N_2_, as the carrier gas, at 8 mL/min.

### *In vivo* experiment

#### Experimental design and diets

On the basis of *in vitro* results, effect of ADY (*S. cerevisiae*) on ruminal fermentation, microbes and serum metabolites of beef cattle were investigated under *in vivo* condition. This trial was designed according to the two-period crossover design (n = 6). It involved two periods, and each one consisted of 15 d for washout period to eliminate carryover impact and 30 d for treatment period. Six adult healthy Xuanhan steers (461.32±18.94 kg) with permanent rumen fistula were divided into two groups randomly and received either basal diet without yeast (CON) or with 0.1% ADY supplementation of total mixed ration DM (ADY). All animals were fed with basal diet (Table 1) twice daily (08:00 and 20:00) in which the ADY was mixed into ration uniformly for ADY group. Daily fed amount of each cattle was determined by feed refusal of the previous day to achieve less than 10% refusal, and water was available in barn for *ad libitum*.

#### Sampling and analysis

- *In vivo* rumen fermentation. Rumen content from each steer was collected at 0, 3, 6, 9, and 12 h after morning feeding through the ruminal cannula on the last day of *in vivo* treatment period (30 d) and filtered by four layers of gauze. pH was measured immediately. Samples were then stored at −20°C for analysis of VFA concentration using the same instrument described above. Ruminal lactate was detected by a commercial kit purchased from Nanjing Jiancheng Biochemical Reagent Co., (Nanjing, China). Another aliquot of rumen fluid was transferred into −80°C for microbial population analysis.
- DNA extraction, PCR primers and real-time polymerase chain reaction (PCR). The ruminal liquid was subjected to bacterial DNA extraction using Bacteria Genomic DNA Extraction Kit (Takara, Dalian, China). Quality of extracted DNA was determined using spectrophotometer (Beckman Coulter Inc., Fullerton, CA, USA) and samples with ratio of OD 260/280 nm above 1.7 were selected for later reaction. The stock DNA was then stored at −20°C in aliquots. The specific PCR primers were synthesized by Huada Gene (Shenzhen, China) and used in this study for amplification of general bacteria, *S. bovis*, *S. ruminantium*, *M. elsdenii* and *Lactobacillus* spp., respectively (Table 2). Real-time PCR assays for enumeration of lactate metabolic bacterial species were performed in triplicate on 96-well plate with BIO-RAD Real-Time PCR detection system (Bio-Rad, Hercules, CA, USA) using SYBR Premix Ex Taq (Takara, China). All PCR reaction mixtures contained forward primer 0.4 μL, reverse primer 0.4 μL, SYBR Premix 5 μL, DNA template 0.8 μL and DEPC-H_2_O 3.4 μL. The values of cycle threshold (Ct) after real-time PCR were utilized to quantify different microbial populations. The relative proportions of these microbes were expressed as a ratio to total rumen bacterial 16S rDNA according to the equation: relative quantification (%) = 2^−(Ct Target − Ct Total bacterial)^×100, where each DNA template of our samples reacting with general bacterial primers was recorded and calculated as Ct (total bacterial). A negative control without the template DNA was performed in every real-time PCR assay for each primer. The PCR amplification of the target DNA, including the annealing and extension temperature, was conducted and adjusted following the references in Table 2.
- Blood biochemical profile. At the end of each period (30 d), blood samples from all cattle were collected by jugular vein puncture before the morning feeding. Serum was obtained by centrifugation (10 min, 1,000×g) and stored at −20°C for further analyses. Concentrations of glucose, triglycerides (TG), total protein (TP), blood urea nitrogen (BUN), albumin and globulin were measured by using the automatic biochemical analyzer (Hitachi, Tokyo, Japan). The non-esterified fatty acid (NEFA) assay kit was purchased from Nanjing Jiancheng Biochemical Reagent Co. (Nanjing, China) for measurement of NEFA.

### Statistical analyses

Data of *in vitro* study was analyzed by one-way analysis of variance using SAS 9.4 software (SAS Institute, Inc. Cary, NC, USA) to determine the effect of ADY supplementation. Responses to increasing levels of ADY (linear and quadratic) were examined using orthogonal polynomial contrast. Duncan’s multiple range tests were performed to test the differences among treatments, which were denoted by different letter superscripts. For the *in vivo* data, individual steers were regarded as an experimental unit, and to compare the difference between CON and ADY, two sample t-test (SAS PROC TTEST) was performed using SAS software on variables of *in vivo* experiment. Significant differences were declared at p<0.05.

## RESULTS

### *In vitro* fermentation characteristics

**Figure 1:**
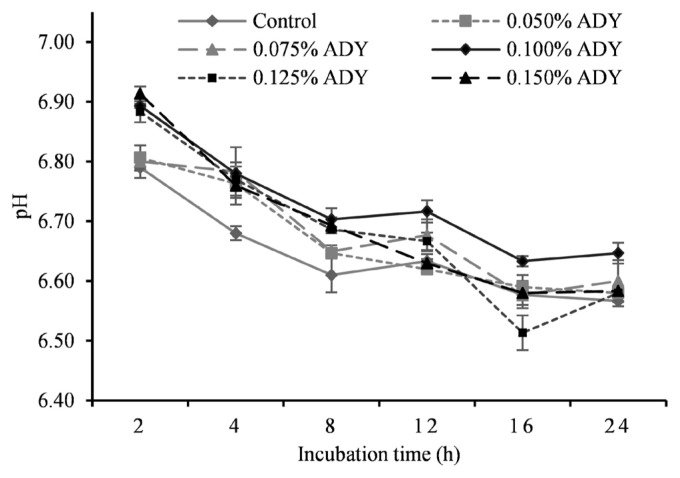
Effect of active dried yeast at different levels on pH of *in vitro* ruminal fermentation. Control, no yeast supplementation; ADY, active dried yeast (*Saccharomyces cerevisiae*) supplementation. 200 mg total mixed ration with 0%, 0.05%, 0.75%, 0.1%, 0.125%, 0.15% ADY of substrate dry matter were fermented *in vitro* for 24 h. Error bars represent standard error.

Table 3 illustrates the detail of *in vitro* fermentation characteristics at different ADY inclusion levels. We observed a significantly greater pH in response to 0.1% ADY addition compared to other treatments. The dynamic change of incubation pH is presented in Figure 1 where significant treatment effect appeared at 2, 4, 8, 16 h. There was a significant treatment effect of dietary ADY on 24 h GP (p<0.01), and those of 0.1% and 0.125% ADY reached the greatest value among treatments. Ammonia nitrogen (NH_3_-N) concentration deceased till 0.1% ADY and then increased quadratically (p< 0.01), where the lowest concentrations were observed in 0.075% and 0.1% ADY groups. Meanwhile, with the increasing of ADY supplementation, MCP in fermentation medium at 24 h incubation was increased and then decreased at high levels (p<0.01), and when 0.1% ADY was added, the highest MCP concentration was observed among the treatments. For individual VFA, acetate undulated along with increasing ADY levels (p<0.01) which also caused linear and quadratic enhancement of butyrate concentration (p<0.01). Peak levels both appeared at 0.1%, 0.125%, and 0.15% ADY levels with no significant difference among them, and that of control exhibited the lowest point. A significant propionate improvement by increasing ADY supplementation (p<0.01) resulted in linear and quadratic reductions in acetate-to-propionate ratio (p<0.01). The ratio dropped to the bottom at dosages of 0.1%, 0.125%, and 0.15% ADY. Total VFA in incubation medium was increased significantly with ADY supplementation levels (p<0.01).

### *In vivo* ruminal fermentation and microbes

#### In vivo ruminal fermentation characteristics

**Figure 2:**
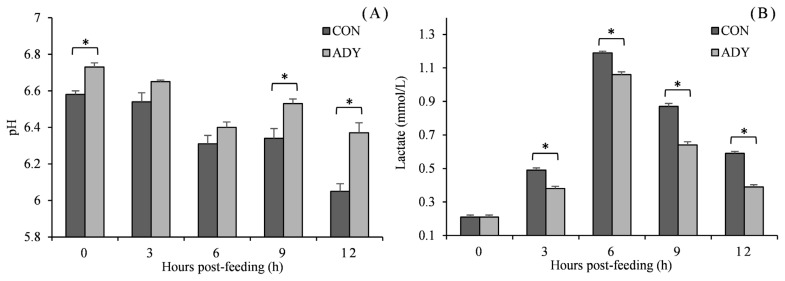
Influence of dietary active dried yeast supplementation on pH (A) and lactate (B) concentrations of rumen fermentation in beef cattle. CON, control, ADY, active dried yeast (*Saccharomyces cerevisiae*) supplementation. Rumen contents were sampled from ruminal cannula at 0, 3, 6, 9, and 12 h post-feeding on 30 d of treatment period. Error bars represent standard error and values at the same time point with “*” differ significantly (p<0.05).

**Figure 3:**
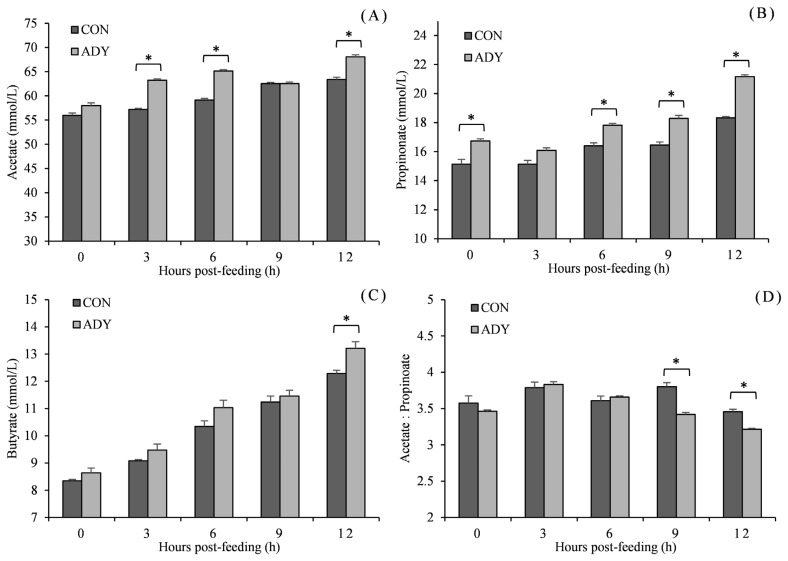
Influence of dietary active dried yeast supplementation on VFA concentration of rumen fermentation in beef cattle. CON, control, ADY, active dried yeast (*Saccharomyces cerevisiae*) supplementation. (A) Acetate, (B) propionate, (C) butyrate, (D) acetate-to-propionate ratio. Rumen contents were sampled from ruminal cannula at 0, 3, 6, 9, and 12 h post-feeding on 30 d of the treatment period. Error bars represent standard error and values at the same time point with “*” differ significantly (p<0.05).

Being consistent with *in vitro* fermentation pH, *in vivo* ruminal pH reduced with time after feeding and reached the lowest point at 12 h in a range from 6.05 to 6.73 (Figure 2). ADY supplementation increased the ruminal pH, and significant differences (p<0.05) were observed at 0, 9, and 12 h in contrast to control diet. Furthermore, lactate concentration increased till 6 h post-feeding and then decreased. Cattle that received ADY had a significantly lower level of ruminal lactate since 3 h after feeding (reduced by 22.5%, 10.8%, 26.4%, and 33.9%, respectively, p<0.05). In the view of VFA (Figure 3), acetate, propionate and butyrate accumulated along with fermentation time. The acetate concentration for ADY supplemented beef cattle was improved significantly at 3, 6, and 12 h post-feeding (p<0.05), meanwhile, propionate concentration in ADY supplemented group was also higher at 0, 6, 9, and 12 h (p<0.05). No significant impact of ADY on butyrate content was observed until 12 h after feeding in the present trial. In addition, with elevated propionate concentration by ADY, the acetate-to-propionate ratio became significantly lower at 9 and 12 h than those in the control diet (p<0.05).

#### Microbial populations

**Figure 4:**
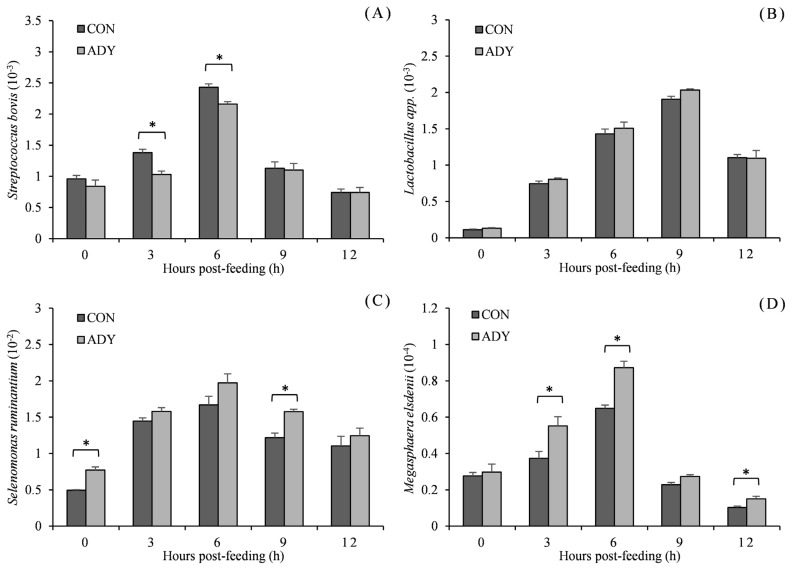
Effect of dietary active dried yeast supplementation on rumen microbial population of beef cattle (ratio to total bacterial 16S rDNA). (A) *Streptococcus bovis*, (B) *Lactobacillus* spp., (C) *Selenomonas ruminantium*, (D) *Megasphaera elsdenii*. CON, control, ADY, active dried yeast (*Saccharomyces cerevisiae*) supplementation. Rumen contents were sampled from ruminal cannula at 0, 3, 6, 9, and 12 h post-feeding on 30 d of treatment period. Error bars represent standard error and values at the same time point with “*” differ significantly (p<0.05).

The microbial populations related to ruminal lactate metabolism in the presence of dietary ADY administration are presented in Figure 4. The populations of microbes relative to total bacteria fluctuated after feeding. They peaked at 6 or 9 h post-feeding and then reduced. *S. bovis* population (ratio to total bacterial 16S rDNA) of beef cattle which received 0.1% ADY supplementation had lower population after feeding and significant difference at 3 and 6 h (p<0.05). However, there was no difference in *Lactobacillus* spp. population for ADY supplementation when compared to the control diet (p>0.05) during 12 h post-feeding. ADY induced an increase of the relative population of *S. ruminantium*, and significant differences between ADY and control diet were witnessed at 0 and 9 h (p<0.05). *M. elsdenii* population was significantly higher in ADY group at the middle and end of sampling time points (3, 6, and 12 h, p<0.05).

### Serum biochemical profile

For serum metabolites in Table 4, the results showed no significant effect on serum TG concentration between ADY and control diet. Cattle given dietary 0.1% ADY had a tendency towards higher cholesterol level (p = 0.09) than those given control diet, and ADY supplementation did not alter NEFA in serum. Following this, blood glucose for beef cattle consuming dietary ADY significantly increased by 21.7% on 30 d (p = 0.01). Regarding BUN, ADY supplementation exhibited significantly reduced level (p = 0.01), and greater TP than those in the control on 30 d (p = 0.02). Albumin concentration of ADY cattle increased significantly (p = 0.04); meanwhile, globulin also tended to be greater (p = 0.07). Besides the improvement of both albumin and globulin, no effect on the albumin-to-globulin ratio occurred with ADY supplementation.

## DISCUSSION

With different ADY dosages, *in vitro* fermentation GP fluctuated and reached zenith at 0.1% and 0.125% levels. Opsi et al [20] observed an increase in fermentation GP by live yeast supplementation, however, in contrast to no effect of inactive yeast. Our results, being in good agreement with the previous investigation, provided potential evidence that live yeast addition conduces to better bacterial activity. It thereby strengthens pyruvic acid-acetate metabolic pathway in ruminal carbohydrates fermentation and produces a rise in CO_2_ production. A negative impact on fermentation GP of high ADY levels was also evident from this study. The reduction could be partly associated with decreased acetate and its metabolism byproducts CO_2_ and H_2_ during carbohydrate degradation [21]. On the other hand, the reason for the lower GP at 0.15% ADY level may be the reduction of methane production. As reported by Tristant and Moran [22], feeding live yeast reduced methane emissions of lactating dairy cows by 4%. When excess ADY supplementation was added in a transition diet, ruminal protozoa, which *Methanogens* lives on and within and are closely related to methane production, were suppressed [23]. Further investigation is needed to fully understand the effects of ADY on ruminal methanogenesis.

The *in vitro* NH_3_-N and MCP concentrations were reduced and increased around 0.1% dosage of ADY, respectively. The finding on decreased NH_3_-N is in line with previous investigation on sheep of Diaz et al [24]. Thrune et al [25], however, did not find any changes in NH_3_-N concentration by live yeast possibly owing to relatively lower supplementation of yeast (0.5 g/head/d) in that study. The lower NH_3_-N concentration could be ascribed to the stimulatory effect of ADY on microbial activities and nitrogen utilization efficiency. Accordingly, MCP synthesis increased at 0.1% ADY level and contributed to greater microbial protein flow to the small intestine. Tripathi and Karim [26] demonstrated higher MCP in lambs in response to live yeast supplementation. It is suggested that bacterial growth factors, for instance, amino acid, vitamins, organic acids of live yeast cells could stimulate autochthonous microbes proliferation and growth [4]. 0.1% ADY adding level brought about the lowest NH_3_-N and maximum MCP concentration. Nevertheless, an over-dose influence appeared afterward, for the possible reason that superfluous live *S. cerevisiae* cells compete for substrate utilization, and releases competitive peptides leading to reduction in bacterial activities [27].

As a vital element influencing ruminal environment and metabolism, pH undulates with feeding and ration structure. Abnormal ruminal pH that comes from ruminal acid metabolism dysfunction has been verified to negatively affect microbial structure, and cause life-threatening metabolism disorders [8]. In the present experiment, the ruminal pH decreased during 12 h of *in vitro* fermentation or feeding time with rapid degradation of easily degradable carbohydrate substrates into acids accumulation (VFA, lactate). Moreover, no difference at low dosages of ADY was observed in current study. When adequate dosage of ADY was added, 0.1% level resulted in noticeable improvement on ruminal pH to stabilize rumen environment for microorganisms. The present results were supported by the investigation of Marden et al [28] where live yeast were supplemented at 5 g/head/d, and this is contrary to Geng et al [29] wherein ADY at 0.8 g/head/d did not change ruminal pH in finishing bulls. The mechanism involved in ruminal pH stabilization by ADY was further investigated hereinafter.

Regarding ruminal VFA, ADY supplementation elevated acetate, propionate, and butyrate concentrations significantly during *in vitro* fermentation study and *in vivo* feeding trial. The increased propionate and butyrate concentrations induced by ADY addition at 5 g/head/d in the work on lactating cows [30] coincides well with our findings. Mao et al [31] reported live yeast cells could increase ruminal bacterial population and activities, especially bacteria such as *Fibrobacter succinogenes*, *Ruminococcus albus* in *in vitro* fermentation. These microbes could boost carbohydrate degradation into VFA (acetate, propionate, butyrate), being mirrored by the heightened GP in this study as well. The acetate-to-propionate ratio response to live yeast displayed a significant reduction at higher levels. A similar result was also reported in Pinloche et al [30]. The increase in the level of propionate is an indispensable precursor of gluconeogenesis for ruminants, indicating the conversion to propionic acid type fermentation, feed efficiency and energy transformation improvement by ADY. As a consequence, total VFA was significantly increased and showed optimized microbial fermentation likewise. Newbold et al [32] concluded that respiration-deficient mutant of *S. cerevisiae* did not affect rumen bacteria, and conversely wild-type strain had stimulatory influence depending on oxide consumption by respiratory activity. The stimulation effect of yeast on bacterial activities could be partly linked to better anaerobic environment created by oxygen scavenging of facultative anaerobic yeast [33]. Though a slight reduction of VFA concentrations was observed at 0.125% or 0.15% ADY levels, the negative over-dose effect of ADY was not significant in the *in vitro* study. We speculate that, under the condition of high concentrate ration, the rapid non-structure carbohydrate degradation into VFA was improved at early phase of fermentation by ADY before its excess proliferation.

It is generally accepted that the decline of ruminal pH after feeding is predominantly caused by VFA and lactate production from microbial fermentation. However, some researchers claimed VFA is one of the main causes of lower ruminal pH [34], as ruminal lactic acid remained low in dairy cows suffering from subacute ruminal acidosis [35]. The explanation appears implausible, and it was also proposed that excess lactate accumulation owing to lactic acid metabolic dysbacteriosis plays an essential role in ruminal pH alteration [36]. Along with the temporary rise after feeding, ruminal lactate concentration in ADY group was lower than the control in our study, and the gap became wider with post-prandial time. From our results, besides the VFA improvement, the lower lactate concentration induced by ADY contributed to higher ruminal pH, since the lactate pKa is 3.7 and has a greater contribution to pH reduction compared to pKa 4.7 of SCFAs [37]. This work provides positive evidence that the pH stabilization property of live yeast may be the consequence of alleviated lactic acid accumulation and dissociation in rumen, which is beneficial for maintenance of rumen microbiota environment. And, further, determining how ADY stabilizes ruminal pH through lactate and associated bacteria was one of the objectives of the present study.

*S. bovis*, which has a greater relative abundance in high concentrate rations without forage [38], can ferment dietary starch into lactic acid. The acid-resistant *S. bovis* could conduce to lactate accumulation beyond the detoxifying activities of lactate utilizing bacteria. Because lactate utilizing bacteria may be restrained by low ruminal pH due to their pH sensitivity [39]. And while *S. bovis* started to multiply rapidly at early fermentation stage, yeast induced its inhibition in the current trial. Our findings are in agreement with the study by Malekkhahi et al [40], suggesting a significantly decrease in *S. bovis* population with live yeast supplementation. The mechanism underlying this interaction may be the competition with *S. bovis* for carbohydrate substrates [27]. Prevention of *S. bovis* proliferation by yeast thereby brought about lactate reduction and lower risk of ruminal acidosis. Shu et al [41] noted that cattle immunized with two lactic acid producing bacteria vaccine had markedly declined lactate concentration and higher ruminal pH, implying the vital capability of *Lactobacillus* spp. and *S. bovis* in lactate generation [42]. In view of the above, the contribution of live yeast to another lactate producing bacteria, *Lactobacillus* spp., in post-prandial rumen fermentation of beef cattle was evaluated, however no significant changes was evident in our results. An experiment into repeated acidotic challenges with or without yeast by Silberberg et al [43] also showed no significant difference of *Lactobacillus* spp. This may be ascribed to greater ruminal pH. Because *Lactobacillus* spp. which has a considerable role as a ruminal lactate producer can not only survive but be dominant at lowered pH (below 5.5) [44].

Furthermore, Wang et al [45] demonstrated significant proliferation in *S. bovis* with concomitant reductions of *S. ruminantium* and *M. elsdenii* populations with high-concentration diet accounted for the sudden ruminal pH drop. Under normal physiological conditions, lactate in rumen remains compatible with efficient ruminal environment (<10.0 mmol/L) [46], and regulated by lactolytic microbes dominated by *S. ruminantium* and *M. elsdenii*. With the aim at boosting rumen function, a study on the impact of *S. cerevisiae* on *S. ruminantium* and *M. elsdenii* in Holstein cows revealed an unaltered result [40]. Our data confirmed the post-prandial stimulation character of *S. cerevisiae* on *S. ruminantium* and *M. elsdenii* growth. Particularly, the significant differences between ADY and control diet appeared at 9 h post-feeding for *S. ruminantium* and 3 to 6 h for *M. elsdenii* when the ruminal lactate accumulated and reached relatively higher level. A positive connection was also observed in a coculture of *S. cerevisiae* and *M. elsdenii*, wherein the bacterial specific activity of lactate consumption was boosted [47]. Pinloche et al [30] reported that the higher rumen pH and less lactate concentration induced by live yeast, were similarly accompanied by an increase in lactate consuming bacterial genera (*Megasphaera* and *Selenomonas*). This gives good agreement with the present study. Our findings on the microbes related to lactate utilization clarified that ADY addition to ruminants is beneficial for growth of *S. ruminantium* and *M. elsdenii*, and more importantly, their metabolic pathway from ruminal lactate to VFA, CO_2_, and H_2_ [48].

We did not find significant influence of ADY on blood TG, cholesterol and NEFA concentrations, all of which are involved in lipid metabolism. Our results were consistent with the works by Yalcin et al [49] and Stella et al [15] showing no alteration in these energy metabolic-related indices of dairy cows and goats in response to *S. cerevisiae* supplementation. More specifically, an increasing tendency towards blood cholesterol was unveiled with live yeast addition, for the possible reason that greater VFA had a contribution to a shift in blood lipid profile [50]. The increased blood glucose by ADY supplementation is in line with a recent study in crossbred Friesian calves of Hassan et al [51]. As an indicator of utilization efficiency of carbohydrates and supplying primary energy source to body cells, glucose is principally synthesized through hepatic gluconeogenesis in ruminants. The enhanced glucose levels of cattle receiving live yeast demonstrated a better energy metabolic status. This might derive from improved rumen fermentation and greater propionate production. Additionally, from our results dietary yeast supplementation caused a significant fall in BUN concentration. It agrees with beneficial role of yeast in lowering the serum urea level in dairy cows [52]. As described by Depeters and Ferguson [53], there is a positive correlation between ruminal NH_3_-N and BUN. This inference appears to be well substantiated by our previous findings on ruminal NH_3_-N and MCP. The decline in BUN with the addition live *S. cerevisiae* implicates a possible mechanism of improved rumen nitrogen capture ability, its conversion to microbial protein from bacterial activity and higher protein outflow from the rumen. Accordingly, an increase in the level of serum TP and albumin in beef cattle fed with 0.1% active yeast was obtained in our study. Hassan et al [51] also observed a linear effect of yeast supplementation in the ration on serum TP of calves. From these data, they had elucidated more insight on the improvement of nitrogen utilization and nutritional status for producing as supported by dietary yeast supplementation.

## CONCLUSION

Active dried yeast supplementation (*S. cerevisiae*) in beef cattle ration boosted *in vitro* microbial fermentation dose-dependently. When ruminal characteristics were considered collectively, the optimal response of ruminal fermentation appeared at 0.1% level of yeast addition. Moreover, our data provide further evidence that dietary live yeast favored a steady ruminal pH mainly relying on the decrease of lactate concentration, rather than VFAs, which is mediated by suppressing the ruminal lactate producing bacteria and stimulation towards lactate consuming bacteria. As a consequence of the optimized rumen environment and fermentation, live yeast supplementation improved the nutritional and physiological condition of beef cattle, including blood glucose and nitrogen metabolism.

## Figures and Tables

**Table 1:** The composition and nutrient contents of diet (air-dry basis)

Items	Proportion
Ingredient composition (%)	
Corn	27.53
Wheat bran	12.13
Distilled grain	21.52
Alfalfa meal	17.48
Rice straw	19.56
NaCl	0.50
NaHCO_3_	0.58
Premix^1)^	0.70
Total	100.0
Nutrient composition	
DM (%)	91.27
CP (%)	11.56
NEmf (MJ/kg)	6.24
NDF (%)	39.35
ADF (%)	24.92
EE (%)	2.43
Ash (%)	7.17
Ca (%)	0.43
P (%)	0.31

DM, dry matter; CP, crude protein; NEmf, combined net energy; NDF, neutral detergent fiber; ADF, acid detergent fiber; EE, ether extract.

1) Premix provided the following for per kg of diet: Vit A, 2,200 IU; Vit D, 2,751 IU; Vit E, 11 IU; Cu, 10 mg; Fe, 50 mg; Zn, 30 mg; Mn, 20 mg; I, 0.5 mg; Se, 0.1 mg; Co, 0.1 mg.

**Table 2:** Specific bacterial primers sequences used for real-time polymerase chain reaction

Target bacterial	Primer sequences (5 to 3′)^1)^	Primer efficiency (%)^2)^	Tm (°C)	Reference
Total bacterial	F: CGGCAACGAGCGCAACCC	97.2	55.3	Denman and McSweeney [54]
	R: CCATTGTAGCACGTGTGTAGCC			
*Streptococcus bovis*	F: TTCCTAGAGATAGGAAGTTTCTTCGG	103.1	55.3	Stevenson and Weimer [55]
	R: ATGATGGCAACTAACAATAGGGGT			
*Selenomonas ruminantium*	F: TGCTAATACCGAATGTTG	94.5	63.4	Bekele et al [56]
	R: TCCTGCACAAGAAAGA			
*Megasphaera elsdenii*	F: GACCGAAACTGCGATGCTAGA	98.8	63.4	Ouwerkerk et al [57]
	R: CGCCTCAGCGTCAGTTGTC			
*Lactobacillus* spp.	F: GAGGCAGCAGTAGGGAATCTTC	101.4	57.7	Qi et al [58]
	R: CAACAGTTACTCTGACACCCGTTCTTC			

1) F, forward; R, reverse.

2) Primer efficiency was calculated referring to Vitti et al [59].

**Table 3:** Effect of active dried yeast at different levels on in vitro fermentation characteristics for 24 h

Items	Supplemental levels of ADY (% DM)^1)^						SEM	p-value		
	
	0	0.05	0.075	0.1	0.125	0.15		Treatment	Linear	Quadratic
pH	6.57^b^	6.58^b^	6.60^b^	6.65^a^	6.58^b^	6.58^b^	0.01	0.05	0.34	0.27
Gas production (mL)	55.3^b^	55.0^b^	53.3^b^	62.1^a^	62.0^a^	52.0^b^	1.06	<0.01	0.55	0.45
NH_3_-N (mg/L)	170.5^c^	171.9^b^	167.8^d^	166.8^d^	169.5^c^	176.2^a^	0.55	<0.01	0.12	<0.01
MCP (mg/100 mL)	16.8^c^	18.3^b^	18.0^b^	19.1^a^	15.1^d^	18.4^b^	0.24	<0.01	0.86	0.31
VFA										
Acetate (mmol/L)	50.7^d^	51.3^cd^	52.3^bc^	53.2^ab^	53.8^a^	53.0^ab^	0.24	<0.01	<0.01	<0.01
Propionate (mmol/L)	14.9^c^	13.0^d^	14.2^c^	16.8^b^	17.8^a^	18.1^a^	0.34	<0.01	<0.01	<0.01
Butyrate (mmol/L)	5.91^b^	6.33^b^	6.11^b^	7.38^a^	7.36^a^	7.35^a^	0.14	<0.01	<0.01	<0.01
Acetate: propionate	3.42^b^	3.94^a^	3.69^a^	3.17^bc^	3.03^c^	2.93^c^	0.07	<0.01	<0.01	<0.01
Total VFA (mmol/L)	71.5^d^	70.7^cd^	72.6^c^	77.4^b^	79.0^a^	78.4^ab^	0.60	<0.01	<0.01	<0.01

ADY, active dried yeast (*Saccharomyces cerevisiae*); DM, dry matter; SEM, standard error of the mean; NH_3_-N, ammonia nitrogen; MCP, microbial crude protein; VFA, volatile fatty acids.

1) *In vitro* fermentation substrates were total mixed ration with 0%, 0.05%, 0.75%, 0.1%, 0.125%, 0.15% ADY of substrate DM.

a–d Values in a same row with different superscripts differ significantly (p<0.05).

**Table 4:** Effects of dietary active dried yeast supplementation on serum metabolites^1)^ of beef cattle (*in vivo*)

Variable	Treatment^2)^		SEM	p-value

	CON	ADY		
Triglycerides (mmol/L)	0.37	0.38	0.03	0.89
Cholesterol (mmol/L)	3.15	3.32	0.05	0.09
NEFA (μmol/L)	299.64	303.94	4.87	0.68
Glucose (mmol/L)	4.05	4.93	0.21	0.01
BUN (mmol/L)	5.00	3.98	0.24	0.01
TP (g/L)	75.13	86.17	2.83	0.02
Albumin (g/L)	30.07	34.03	1.05	0.04
Globulin (g/L)	45.07	52.13	2.02	0.07
A/G	0.67	0.66	0.02	0.79

SEM, standard error of the mean; NEFA, non-esterified fatty acid; BUN, blood urea nitrogen; TP, total protein; A/G, albumin-to-globulin ratio.

1) Blood samples were collected from the jugular vein before morning feeding on 30 d of treatment period.

2) CON, control; ADY, active dried yeast (*Saccharomyces cerevisiae*).
